# Current Trends in Understanding and Managing Equine Rhodococcosis

**DOI:** 10.3390/ani10101910

**Published:** 2020-10-18

**Authors:** Alicja Rakowska, Anna Cywinska, Lucjan Witkowski

**Affiliations:** 1 Department of Veterinary Epidemiology and Economics, Institute of Veterinary Medicine, Warsaw University of Life Sciences, Nowoursynowska 159c, 02-787 Warszawa, Poland; lucjan_witkowski@sggw.edu.pl; 2 Department of Pathology and Veterinary Diagnostics, Institute of Veterinary Medicine, Warsaw University of Life Sciences, Nowoursynowska 1, 02-787 Warszawa, Poland; anna_cywinska@sggw.edu.pl

**Keywords:** *Rhodococcus equi*, rhodococcosis, foals’ pneumonia

## Abstract

**Simple Summary:**

Pneumonia caused by soil bacteria *Rhodococcus equi* occurs in the foals of most horse breeds all over the world, posing a significant challenge for veterinary practitioners. For this reason, many researchers constantly try to find new solutions for successful prevention and management of the disease, but it still remains poorly controlled. This paper presents some promising ideas published during the last decade. Several strategies mentioned below have already been introduced to clinical practice like a variety of immune stimulators, but many others are still under academic considerations. The compilation of these materials may help to understand the complexity of the problem and show the directions for effective practice in the future.

**Abstract:**

The aim of this review was to summarize studies on equine rhodococcosis over the last decade. For many years *Rhodococcus equi* has remained one of the major health challenges in the equine breeding industry worldwide. Recently, many novel approaches and ideas have been described and some of them were initially implemented into the clinical practice. This study reviews a variety of new information about neonatal susceptibility, clinical appearance, considered and applied diagnostic procedures and treatment alternatives, factors limiting accurate prognosis, ideas regarding environmental management and prophylaxis considerations. Although multiple research were conducted, the main problems such as high morbidity and mortality, a lack of reliable prevention strategies and treatment limitations are still unresolved and require further scientific effort.

## 1. Introduction

Rhodococcosis is one of the major causes of health problems in foals before weaning. Its etiological agent, a well-known, ubiquitous, opportunistic, intracellular soil saprophyte, *Rhodococcus equi,* may be responsible for severe pyogranulomatous bronchopneumonia often leading to deaths of affected foals. Morbidity rates may exceed 40% [1,2] on some farms, but the problem remains strictly endemic and as such in other places it may never occur. Relevant costs of the disease include the high expenses of prevention, antimicrobial treatment and management of the patients but are also suspected to lower probability of successful performance in adulthood [1,3] and finally a loss of foals [4]. Rhodococcal pneumonia presents a clinical and scientific challenge and many aspects still remain unclear. This study is focused on current trends and recently reported solutions aimed to improve the management of equine rhodococcosis.

## 2. Pathogenesis and Virulence

Up to now the virulence of *Rhodococcus equi* is believed to be related mainly with 90 kb virulence associated plasmid (VAP), which contains pathogenicity islands (PAIs) considered responsible for its pathogenic potential [5]. Environmental strains of *Rhodococcus* often do not contain Vaps unlike most of those obtained from clinical patients [6,7]. Among 20 different proteins encoded on PAIs, 6 determine the virulence of a certain strain. Only one of those proteins, virulence associated protein A (*VapA*) for many years has been known as connected with intracellular survival in equine lung alveolar macrophages and subsequent development of clinical pneumonia [8,9]. What has been recently discovered is that there are actually three genes encoding proteins required for intracellular survival and growth, hence implicating death of phagocytic cells. Whereas *vapA* had been confirmed to play a central role in rhodococcal virulence, another two genes, *virR* and *virS*, have been revealed to modify chromosomal transcriptome and allow for intracellular parasitism [5].

## 3. Susceptibility

According to epidemiological data, clinical signs occur in 10–20% of foals in endemic farms in the US [10] but ultrasound evidence of pulmonary lesions have been reported to vary between 30 and 60% [4]. Currently, it is mostly accepted that the foals become infected in the very first moments of life, probably days or even hours due to the presence of *R. equi* in soil, manure and in the airborne dust, which is easily inhaled and can cause infection [6,11,12]. This hypothesis was partially confirmed by one of the clinical studies, where the doses and time of highest susceptibility were checked. The results have shown that neonatal foals were the most susceptible for rhodococcal infection and much more likely to develop clinical evidence of pneumonia even after lower doses of bacteria. Older foals, 3 or 6 weeks of age, were already less susceptible and often did not progress to clinical signs after the same low doses of bacteria [13]. These results revised a partially inadequate approach in many previous studies, where older foals, in their 3rd or 4th week of life, were challenged with high doses of bacteria. This experimental protocol resulted in acute and a very severe form of pneumonia, not common in field conditions after natural exposure, where subclinical or chronic pneumonia with many spontaneous regressions predominate. The aforementioned studies confirmed that *R. equi* infection is strictly age and dose dependent. Susceptibility is the highest in neonates and decreases with age, but high doses of bacteria cause the severe, rapidly progressing form of pneumonia and usually subsequent euthanasia, even in older foals [13]. Pulmonary abnormalities were detectable in all challenged animals. Foals that received lower doses (10^2^ cfu or 10^3^ cfu) developed mostly well-defined hypoechoic lesions whereas those challenged with higher doses (10^4^ cfu to 10^6^ cfu) usually presented large areas of consolidated lungs. All lesions seemed to decrease in size over time. Survival probability peaked significantly for foals challenged later in life (3–6 weeks) or for those infected with lower doses [13]. Although foals between 3 weeks and 8 months of age are 14 times more likely to be infected with *R. equi* than any other bacteria [14], mixed bacterial infections are frequent but not yet linked with worse prognosis [15]. Neither genetic factors [16] nor microbiome and fecal metagenome traits have been confirmed to increase susceptibility [17].

## 4. Clinical Signs

Clinically, rhodococcal pneumonia is a form of lower respiratory tract infection. The most common presentation in foals includes fever, cough, increased respiratory rate and heart rate and abnormal tracheal and lung sounds on auscultation. Nasal discharge may occur in some cases. Progression of pneumonia is insidious and pulmonary lesions may already become quite extensive before the clinical signs appear. On rare occasions, the disease may occur as a peracute form, when previously healthy foals develop respiratory distress, sudden in onset and progressing rapidly to death within less than 48 h. Foals may also manifest extrapulmonary signs of infection with *R. equi*. These manifestations usually include diarrhea, colic and weight loss. Since extrapulmonary pathologies are usually linked with higher mortality rate [18,19], an early diagnosis is beneficial and should be recommended to improve reliability of the prognosis and treatment possibilities [19,20]. Other extrapulmonary signs are less common and include aseptic and septic inflammations in the joints and abscessations in the eyes [19] or other tissues [18,21,22]. Unfortunately, the disease is quite frequently confirmed at necropsy. Post mortem examination usually reveals pyogranulomatous pneumonia and abscessation of other tissues in the case of the extrapulmonary form, which may refer to as much as 76% of lost foals, which underwent necropsy according to one paper [18]. In acute cases, foamy exudate in trachea and bronchi is observed indicating severe pulmonary oedema.

## 5. Diagnosis

Rhodococcal pneumonia is usually confirmed by the isolation of virulent type of bacteria from tracheobronchial aspirate (TBA) obtained from a foal presenting clinical signs or positive PCR test and detection of radiographic or ultrasonographic signs of pneumonia [23]. Previously, many methods were recommended for diagnosis, including X-rays [1,24,25,26] or white blood cell count (WBC count) [14,27], however most of them are currently considered ineffective or too labor demanding.

At present, ultrasound is found the easiest and most common for both diagnosis and monitoring of the disease [1,2,28,29,30]. Among the advantages of ultrasound, the safety for the personnel, short time of testing and data interpretation and availability to screen the animals regularly without causing major stress or health risk should be listed. What is more, this method is more sensitive for the assessment of peripheral lung masses or lesions located in the areas superimposed over any other soft tissues, which are hard to assess radiologically. Ultrasound examination was also mentioned to be suspected of greater usefulness in assessing pleural effusion or lymphadenopathy [1]. On the other hand, not all of the lesions are located superficially so some of them may not be recognized with this technique. The most common ultrasound findings in thorax screening are comet tails, reflecting any fluid or cellular infiltration on the pleura. When they are limited in number and occur ventrally, they are considered clinically irrelevant, however an increased amount mid or cranially located comet tails should be interpreted as pulmonary inflammatory disease [31]. On ultrasonographic examination, abscesses are well-defined hypoechoic nodules, variable in size, whereas consolidated lungs are ill-defined, hypoechoic regions sometimes with visible vessels and/or bronchi [13]. Ultrasound is performed successfully by using any transducer 5–7.5 MHz that fits in between the ribs and wetting the ribcage with alcohol, which makes it available to be performed by the vast majority of field veterinarians Clipping is usually not necessary. In healthy patients only the pleura can be visualized due to the reflection of ultrasounds by normally aerated lung parenchyma. Pleura is a thin, highly hyperechoic line that looks like it multiplicated due to air reflection, called the reverberation artifact. Lung parenchyma can only be visualized through an acoustic window when fluid or cellular accumulation occurs just below the pleura. The echogenicity of the lesions depends on the presence of cells, fluid or type of nodule and presence of the capsule [32]. One of the field research programs revealed that over 95% of foals without any clinical signs, from the farm with endemic rhodococcosis developed lesions detected on the ultrasound, including pulmonary abscessation or consolidation, diagnosed within the examination period (3–16 weeks of age). Similarly to the challenge with *R. equi* mentioned above, abscesses’ size decreased with time. Changes were detected predominantly in the 5th and the 7th week of age [2].

The method generally accepted as golden standard in antemortem diagnosis of *R. equi* is to confirm the presence of bacteria in TBA. However, in one paper 36% of foals positive for post-mortem culture and the same percentage of those positive for radiological or ultrasonographic lesions were negative in the PCR test from TBA [33]. Another paper has reported that *R. equi* was cultured only from up to 54% of the foals presenting ultrasonographical signs of pneumonia, checked during the clinical trials. Nevertheless, in the same trial the presence of *R. equi* was confirmed in all 24 lost foals with ultrasonographic lesions [30]. Possibly some foals can be positive in TBA samples due to subclinical infection or inhalation of the pathogen that does not result in any clinical abnormalities [4]. Moreover, TBA sampling has some significant limitations. The procedure is expensive, invasive and requires attendance of stable staff to handle the animals. Foals usually need sedation during the endoscopic examinations, which creates additional risk and cost for the owner and so that it should not be recommended for multiple use as regular monitoring of the disease [2]. The risk may also be unacceptable for the foals with severe respiratory distress. In addition, microbiological culture takes more than 3 days in total to confirm the presence of the pathogen, which is unacceptably long in severe and often acute disease. On the other hand, TBA culture is much more popular in clinics, where the probability of rhodococcal infection is relatively lower than on endemic farms and a well-equipped laboratory is fully available [14].

Basic parameters routinely used in the diagnosis of equine respiratory diseases are of limited importance in rhodococcosis. One of the recent papers analyzed the results of basic tests performed in the foals presenting respiratory signs during hospitalization [14]. The following parameters were taken into consideration: radiological picture, the presence of bacteria in TBA, temperature, abnormalities in the respiratory rate, pulse, hematocrit, log of WBC and neutrophil counts, number of band neutrophils, monocytes, lymphocytes, eosinophils, total protein, globulins and fibrinogen concentrations. Only four of them: WBC count above 20,000 cells/uL, plasma fibrinogen concentration above 700 mg/dL, Gram + coccobacilli in TBA and radiological evidence of pulmonary abscessation were found suggestive but yet not definitive for rhodococcosis, so they cannot be considered as a single diagnostic tool [14].

Even if some parameters, like acute phase proteins (APPs) concentrations are elevated in most clinically affected foals [13], they do not seem clinically relevant. Fibrinogen is one of the major APPs in horses. Its concentration, although non-specific, is frequently measured as an additional and easily available parameter and is suspected to peak more quickly or highly during development of pneumonia in foals than in most of the other conditions, but still it is neither sensitive nor specific enough [14]. Its concentration is considered to be significantly different between healthy and subclinically affected foals during weekly testing. However, due to the lack of standardized physiological values for foals and a low number of animals analyzed up to now, the method requires further studies [34]. As an alternative, serum amyloid A, also belonging to major APPs in horses, has been proposed. It has been suspected to be more sensitive due to its minimal or undetected concentration in healthy animals and high (more than 100 times) peak in inflammatory conditions [34]. Unfortunately, one paper has shown that clinically healthy foals had elevated levels of SAA without any clinical signs while nearly 30% of foals with bacterial bronchopneumonia had normal SAA levels during the test [35], and others found no significant differences between affected and healthy foals [34,36].

Nasal and fecal swabs are widely considered invalid [4,37], however one paper reported that 54.5% of foals presenting subclinical pulmonary findings were positive for *Rhodococcus equi* vapA in either nasal or fecal swabs or both [2]. What is more, the virulent type of bacteria was identified during the occurrence of ultrasonographic changes or even before them. Up to date there are also no blood tests specific to detect rhodococcal pneumonia. Serology tests have been acclaimed as not useful [14,38]. Trials with five different serological assays have shown low sensitivity in the case of a low cut off point and low specificity when the cut-off point was increased [38].

## 6. Treatment

The most important problems in treating *Rhodococcus equi* infections are the limited number of effective antimicrobial drugs (Table 1) and an increasing resistance. Generally, the number of antibiotic groups used in equine medicine is very restricted and antimicrobial resistance has grown significantly over the last decades [39,40,41]. Recommendations for treatment strategy were settled a few decades ago but prior to the 2000s reports on resistance were very rare [42]. A similar problem refers to the treatment of equine rhodococcosis and human tuberculosis, also alike due to its intracellular character [11]. There are very few newly discovered antibiotics in the last decades and new substances from the same group are much more likely to be ineffective due to cross-resistance [30]. When it comes to other antimicrobial groups, less frequently used in horses, most of them produce serious adverse reactions, especially in foals like aminoglycosides (nephrotoxic), fluoroquinolones (arthropathy) or most of tetracyclines (bone deformities) [39]. At last, some drugs for which *Rhodococcus* is generally susceptible (e.g., imipenem and vancomycin [39]) belong to substances that are or should be or may be restricted only for human clinical use [43].

The combination of a macrolide with rifampicin remains the recommended therapy for foals with clinical signs of infection caused by *R. equi* for about 40 years. However, the most recent papers suggest that combining rifampicin with some macrolides like clarithromycin or one of the newest, semi-synthetic macrolide tulathromycin can allow for lowering the effective concentration of rifampicin’s in bronchoalveolar cells [40,44,47]. There are also papers suggesting that the use of macrolide as the only antimicrobial treatment (azithromycin or gamithromycin) may be at least equally effective as a standard combination of antibiotics [4,46]. Nevertheless, this approach is suspected to create more risk of increased antimicrobial resistance than combination with rifampicin [48]. Progress in rhodococcosis therapy for many years has been limited only to the implementation of new macrolides. There were no data that could indicate the preferred antimicrobial agent or agents for foals infected with isolates resistant to macrolides or rifampicin. Recently, a combination of doxycycline and azithromycin has been evaluated as equally effective to the standard combination for the cases with mild or moderate pneumonia [30]. Doxycycline is wildly used in veterinary pulmonary issues and in bronchoalveoli it can reach Minimal Inhibitory Concentration for 90% of microorganisms (MIC_90_) for most bacteria responsible for bronchopneumonia in foals. At last, doxycycline combinations seem to have less adverse effects such as diarrhea than rifampicin [30]. Another alternative recently checked for antirhodococcal potential in foals was liposomal gentamicin, which exceeds MIC_90_ for *Rhodococcus* in bronchoalveolar fluid for over 48 h. Its main disadvantage is a wildly described nephrotoxicity, which considerably shortens the time of safe use. However, long antimicrobial activity in the affected tissues may be a key to modify the administration protocol and its effective use in the future [49].

Other promising results refer to gallium maltolate, a semi metal that mimics ferric iron and can inactivate some iron-dependent enzyme reactions in some bacteria, including *Rhodococcus*, which seems equally effective as a standard antimicrobial therapy for the subclinical form of pneumonia. Unfortunately, this prevention strategy is not commercially available yet [50]. Treatment is generally prolonged, expensive and, what is the worst, sometimes ineffective. Furthermore, the macrolides recommended for *R. equi* infections can cause life-threatening hyperthermia and diarrhea in foals and fatal colitis in mares. Despite many attempts, solid recommendations for the appropriate moment of implementation of antimicrobial treatment are also unknown. Currently there are two methods for the evaluation of lesions: the Slovis score is based on the maximal diameter of the largest lesion [50,51] and the total maximal diameter (TMD) is based on the total diameter of all detected lesions [37]. However, the cut off point for both scores is also not settled and usually used on the basis of individual clinical experience and changes significantly over time [29]. Surprisingly, in the last decade, it has been shown that in the foals with clinical signs and abscess score (based on the total lesions’ diameter) lower than 11 cm (2013–2015) or even 15 cm (2016) the treatment may not be necessary (Table 2). What is more, a lack of treatment in these cases did not increase mortality rates in the corresponding year [29], but a percentage of foals treated with antimicrobials decreased significantly from 81.9% in 2008–2011 to 50.9% in 2012–2016 [29]. Similarly, failures of treatments and recurrences of pulmonary abscess decreased over the mentioned periods. In other studies, one farm was able to decrease antimicrobial use for nearly 80% without increasing mortality rates [45,46].

Early detection of subclinical findings and temporary implementation of an antimicrobial treatment to these foals seemed a valuable method to prevent clinical onset and mortality due to *Rhodococcus* pneumonia. The prophylaxis with macrolides was reported over a decade ago [52]. However, decades of widespread overuse of antibiotics in control and prevention programs implemented at breeding farms created another problem—increasing resistance to macrolides and rifampicin in equine *R. equi* isolates. Many authors have suggested that when ultrasound screening became more popular, the use of antimicrobial preventive treatment increased at many farms, which may contribute to significant increase of antimicrobial resistance during the last twenty years [53,54]. It should be highlighted that in most of the cases spontaneous regression of the lesions occurs without any treatment [4,28,29,44] and may refer even to all foals affected subclinically [2,30,44]. A recent epidemiological study identified that 76% of isolates from the tested farms were resistant to either one or both antimicrobials [42] and showed an alarming increase of nearly 15% during the last 10 years [53]. On the other hand, most of the papers mentioned decrease in the mortality rates due to antimicrobial treatment. It is also not clear if the resistance occurs only on endemic farms or results from increased use of rifampicin and macrolides in human medicine. Regardless of the cause, widespread dissemination of resistance genes in the environment where many other pathogenic bacteria exist is a concern for both animal and human health [53]. Additionally, in one study only 55% of coexisting bacteria were susceptible to erythromycin and/or rifampicin. It creates additional risk of stimulating bacterial resistance in species other than *Rhodococcus*, especially considering the fact that about 65% of the infections reported in this paper were of mixed origin [14]. On the other hand, some research did not find an association between mixed bacteria infection and the severity of radiographic findings [1].

## 7. Prognosis

The prognosis seems to depend partially on the severity of the clinical signs. Some authors reported that survival rate increased from 20% before use of the combined treatment to about 80% currently [18,33]. Generally, a successful treatment is estimated for 60–70% [15,51,55]. There is also one record claiming nearly 100% success when both ultrasound screening and antimicrobial prophylaxis for foals with pulmonary changes were used [56]. Unfortunately, there are no methods to distinguish between the foals that require treatment and spontaneous regressors yet. During radiological evaluation, the presence and number of cavitary lesions and nodules claimed to be helpful in distinguishing survivors and non-survivors [1].

## 8. Environmental Management

Several factors positively correlating with increased bacteria concentration in inhaled air have been suspected to influence the morbidity rates, e.g., density of mares and foals, higher mean temperatures, less soil moisture, reduced grass height or late foaling season. A previously suspected theory that rhodococcosis is more popular or even restricted to areas with warmer climate was generally proven to be wrong [34,47]. In another paper, a correlation between the month of birth and probability of developing subclinical lesions has been explained by environmental reasons [57]. Some practitioners consider lime as a limiting factor due to prolonged influence on maintaining higher soil pH, whereas *Rhodococcus* prefers a more acidic environment [47].

Another interesting hypothesis claims a correlation between dietary management and the severity of some bacterial infections. Preliminary studies revealed that high protein and high carbohydrate diet may lead to development or exacerbation of clinical signs in one of the most popular equine bacterial diseases—strangles [58]. A more recent paper describes the relation between an excessive diet and its influence on the production of cell wall polysaccharides, which are one of the major pathogenicity factors in many bacteria, including *Streptococcus*, *Pasteurella* and *Clostridium* but also *Rhodococcus* [59]. Since the equine gastrointestinal tract and gut microbiota effectively uses food very high in fiber, an overnutritious diet may trigger intestinal dysbiosis and eventually diminish natural immunity [59]. This finding may bring partial explanation of a wide variety of clinical manifestations among different studs and breeding seasons. Unfortunately, no environmental management practice has been reported as effective enough to be strongly recommended for the control of the disease yet [4].

On the other hand, some papers indicate a higher risk of rhodococcal pneumonia in foals exposed to higher air concentrations of pathogens in their first two weeks of life [12]. Moreover, in older foal concentrations of fecal shedding seems higher, which in turn may affect airborne concentrations, posing a significant risk for the younger ones [47]. Another study has shown that after the intratracheal challenge, 3–4 week old foals developed clinical symptoms and *R. equi* shedding increased in both the foals and their mothers, regardless of the dose of inoculation. These results have proven a significant role of infected foals but also their dames in increasing the airborne concentration of *Rhodococcus* in the environment, which may have yet an undetected effect on the prevalence of the disease. What is more, the same study confirmed a significant decrease of bacteria number in equine manure after standard composting for 7 days, which can also be taken into consideration as a future recommendation for environmental practices [60].

## 9. Inhibitory Compounds

The newest techniques of genome sequencing may help to turn antirhodococcal strategies into a new direction. The recent studies have revealed the genes responsible for secondary metabolites, which may not occur in standard laboratory conditions but have strong inhibitory effects on most of the *Rhodococcus* strains tested up to date. Based on the molecular mass the molecular composition was postulated to be C_52_H_78_O_13_. What is more *R. equi* seemed to be particularly sensitive for these newly discovered molecules. Surprisingly, this new substance was detected when selected isolates of soil *Rhodococcus* were grown in the temperatures below 22 °C. Thus, it may pose at least a partial explanation for some field observations regarding a higher prevalence of rhodococcal infections during warmer months or in warmer climates, when inhibitory molecules are not produced. These novel metabolites may be the key to the future environmental management of *Rhodococcus* but require further attention and research [61].

## 10. Prevention Strategies

According to clinical data, there is no prevention strategy effective enough against rhodococcosis, although many methods have been considered [41,52,56,62]. Due to the structure of equine placenta, foals are born immunologically naive and need to gain a maternal antibody via colostrum [63]. Thus, the quality of colostrum, its amount and time of first suckling are absolutely essential for a neonates’ immunity. Prevention of rhodococcal pneumonia can be partially obtained by administration of hyperimmune plasma (HIP), which is a standard procedure in many breeding farms in US [4,57]. It is believed to reduce the severity of pneumonia in foals after the experimental challenge or natural exposure, but it creates some risk due to intravenous administration and it seems not to be fully effective in prevention of developing clinical signs in general. The efficacy of HIP administration is still controversial, as it has not been proven as 100% effective [4]. Commercially available plasma in the US is quite expensive and its quality is not standardized due to individual differences of donor horses and diversity of *Rhodococcus equi* strains in particular areas. Even if general IgG concentrations were similar, specific anti-VapA ones varied among samples [64]. Some of the latest papers also indicated that the effectiveness of this strategy might be dose-dependent, because 2 L of HIP was more beneficial than administration of 1 L or less of the same product [57]. What is more, when two methods: vaccination of mares and administration of HIP were used, clinical sings in the foals occurred later, were less severe and mortality rate was lower [65]. As it has been previously confirmed, clinical signs of pneumonia in foals can be associated with significantly higher fecal shedding of *Rhodococcus equi*. Administration of hyperimmune plasma has become a recommended solution despite its poorly understood mechanisms of action, because it decreased the severity of the clinical signs of pneumonia, but also seemed to decrease the overall risk by lowering the level of shedding by affected foals [64].

Vaccinations against *R. equi* are wildly awaited and often considered a form of prophylaxis. Unfortunately, despite many trials none of them was claimed effective enough to become commercially available [65]. Up to now there are no vaccinations available neither for mares nor for the foals [62]. In some countries different types of autogenous vaccines are used [66]. Many different methods have been used for mares and foals immunization thus far and some of them provided even encouraging results during preliminary study on a small research group. A promising method of inactivation of *Rhodococcus* using an electron beam in the foal vaccine has been recently proposed. The eBeam inactivation allows the maintenance of the outer cell wall integrity crucial in promoting the immune response [62,67]. Unfortunately, there were neither significant improvement in regard to the severity and durability of the infection nor desirable changes in both systemic and mucosal immune responses [37]. However, immunization of mares with the eBeam vaccine seems worthy to consider in the future.

Another recent study concerned a vaccine based on highly conserved microbial surface polysaccharide, which is expressed on some intracellular pathogens including *Rhodococcus*. To promote the cell mediated response, which was suspected to be protective in foals, the researchers tried to vaccinate mares against poly-N-acetyl glucosamine (PNAG). Most foals from vaccinated dames did not develop clinical signs of the disease whereas unvaccinated dams gave birth to foals, which the majority developed pneumonia after being challenged with *Rhodococcus equi* in the 4th week of life. Foals from vaccinated mares presenting clinical signs had also shorter and less severe clinical onset and smaller and fewer ultrasonographic lesions. The major weakness of this study design was challenging foals at 25th–28th days of age, which is too late to mimic the naturally occurring infection [68]. What is more, foals challenged after administration of hyperimmune plasma from mares vaccinated against PNAG that would mimic colostral antibody transfer presented similar results and seemed to be more resistant to the disease and also did not present clinical sings whereas the control group of foals that received normal plasma developed signs of pneumonia.

## 11. Conclusions

*Rhodococcus equi* infection in foals is a very well known and widely described problem, but there is still a lot of significant information missing. Hence, new ideas are still welcome and carefully examined as a potential key factor responsible for proper solutions. Since the majority of current recommendations are not completely satisfying in the field, further research is extremely necessary. Considerations regarding risk factors and predispositions, immunoprophylaxis, especially effective vaccinations or immunostimulants like HIP and, at last, methods of successful management and treatment are likely to be the main scientific goals for the future.

## Figures and Tables

**Table 1:** Antibiotics recommended in equine rhodococcosis.

Active Substance	Dose	Duration of Recommended Treatment	Reference
Rifampicin Erythromycin	5 mg/kg PO 2/daily 25 mg/kg 3/daily	4–9 weeks	[33]
Rifampicin Clarithromycin	5 mg/kg PO 2/daily 7.5 mg/kg 2/daily	3–12 weeks	[4]
Rifampicin Azithromycin	10 mg/kg PO daily 10 mg/kg PO daily	6 weeks	[29]
Doxycycline in monotherapy	10 mg/kg PO 2/day	6 weeks	[44]
Doxycycline Azithromycin	10 mg/kg PO 2/day 5 mg/kg PO daily	6 weeks	[30]
Tulathromycin in monotherapy	2.5 mg/kg IM 1/week	6 weeks	[45]
Gamithromycin in monotherapy	6 mg/kg IM 1/week	6 weeks	[46]

**Table 2:** Changes in size of the lesion recommended for treatment over time.

Size of the Lesion	Year	Reference
≥10 mm *	2004	[51]
≥21 mm *	2005	[51]
≥80 mm ^	2012	[29]
≥100 mm ^	2014	[29]

*—according to the Slovis score; ^—according to the total maximal diameter (TMD).
